# ExoShorkie: predicting RNA-seq coverage of exogenous genomes in yeast by transfer learning

**DOI:** 10.1093/bioinformatics/btag369

**Published:** 2026-08-21

**Authors:** Jonathan Mandl, Yaron Orenstein

**Affiliations:** Department of Computer Science and Artificial Intelligence, Bar-Ilan University, Ramat Gan, 5290002, Israel; Department of Computer Science and Artificial Intelligence, Bar-Ilan University, Ramat Gan, 5290002, Israel; The Mina and Everard Goodman Faculty of Life Sciences, Bar-Ilan University, Ramat Gan, 5290002, Israel

## Abstract

**Motivation:**

Predicting the RNA-seq coverage of native and exogenous sequences is central to many molecular- and synthetic-biology applications. Substantial progress has been made in developing methods to predict the RNA-seq coverage of native genomic sequences, with the recently developed Shorkie achieving state-of-the-art performance in yeast. However, prediction performance of these methods over exogenous DNA is still unknown. Recent studies measured RNA-seq coverage of large exogenous genomes in yeast, providing a unique opportunity to train machine-learning models on a large exogenous sequence space and to improve both prediction performance and our understanding of regulatory mechanisms.

**Results:**

We introduce ExoShorkie, a method we developed by extending Shorkie through transfer learning across multiple exogenous RNA-seq datasets. We demonstrate that ExoShorkie significantly improves prediction performance on held-out exogenous genomes and outperforms both a native-genome-trained Shorkie baseline and Yorzoi, the only competing method for predicting exogenous RNA-seq coverage in yeast, in cross-validation and in leave-one-genome-out evaluations. Furthermore, through interpretability analyses we reveal biologically meaningful regulatory motifs and distinct regulatory rules in exogenous genomes in yeast, providing new insights into transcriptional regulation.

**Availability and implementation:**

ExoShorkie is available at https://github.com/OrensteinLab/ExoShorkie.

## 1 Introduction

Models that can reliably predict RNA-seq coverage directly from DNA sequence hold promise for enabling the design of synthetic sequences with desired expression levels across a wide range of biotechnological and therapeutic applications. Recent advances in high-throughput genomics and deep learning have driven the development of models that achieve high predictive performance in mapping genomic sequences to gene expression. These models, including Enformer and Borzoi, commonly employ transformer-based neural networks to predict RNA-seq coverage and related functional tracks from DNA sequence at high resolution (Avsec *et al.* 2021, Linder *et al.* 2025).

Despite the high predictive performance these models achieve on native genomic sequences, they have been shown to generalize poorly to out-of-distribution synthetic sequences involving rearrangements of regulatory elements (Ribeiro-dos-Santos and Maurano 2025). High predictive performance on such tasks is essential for the design of complex synthetic sequences with multiple regulatory elements. However, native genomes lack the sequence diversity required for models to learn the full complexity of the gene-regulatory grammar (de Boer and Taipale 2024). Consequently, training beyond native genomic sequences may be required to learn complex regulatory rules. Randomized DNA has been proposed as a potential solution to enable models to be trained on a much larger sequence space while reducing the biases and homology inherent to native genomes (de Boer and Taipale 2024).

While deep-learning models can successfully predict the expression of random promoter sequences in massively parallel reporter assays (de Boer *et al.* 2020, Vaishnav *et al.* 2022), these models focus on isolated regulatory elements within short reporter constructs. Consequently, they cannot account for multiple regulatory elements acting jointly within a gene-regulatory architecture (Zrimec *et al.* 2020). Computational methods thus remain limited in their ability to predict the genome-scale function of complex synthetic constructs (James *et al.* 2025). Training on long random DNA integrated within a chromosomal locus can provide models with examples beyond the native genome of multiple regulatory elements acting together, as well as locus-level mechanisms, such as 3D looping, insulation, and competition (de Boer and Taipale 2024). However, generating long random DNA sequences and inserting them into living cells has until recently been considered a highly challenging task. Only recently, experimental techniques have advanced to produce complete synthetic genomes and insert them into living cells (James *et al.* 2025). As a result, multiple studies measured the expression of long exogenous DNA sequences in yeast, which can serve as a proxy for the vast space of out-of-distribution DNA (Zhou *et al.* 2022, Camellato *et al.* 2024, Luthra *et al.* 2024, Meneu *et al.* 2025).

Recent efforts have begun to explore genome-wide RNA-seq coverage prediction in yeast. Shorkie was developed as a transformer-based DNA language model pretrained with a self-supervised objective on diverse fungal genomes and fine-tuned to predict thousands of RNA-seq tracks in yeast (Chao *et al.* 2025). However, it was trained solely on native genomic sequences and has not been evaluated on exogenous genomes. In contrast, Yorzoi was developed as a similar transformer architecture to predict RNA-seq coverage on exogenous human DNA sequences in yeast (Schneider *et al.* 2025). While the Yorzoi study demonstrated that exogenous DNA can be modeled, it remains unclear whether models trained on one exogenous genome can generalize across other exogenous genomes. Moreover, none of these studies provided interpretability analyses of the regulatory logic captured in exogenous DNA. Addressing these limitations is essential for evaluating both the generalization ability of such models and the regulatory rules they learned.

Here, we present *ExoShorkie* to accurately predict the RNA-seq coverage of exogenous genomes in yeast. We first demonstrate that the original Shorkie model, when trained exclusively on native genomic sequences, fails to generalize to exogenous DNA. To address this, we developed ExoShorkie, a model trained by fine-tuning Shorkie on RNA-seq datasets of exogenous DNA sequences in yeast. We show that ExoShorkie outperforms both a native-genome-trained baseline and the competing exogenous DNA approach, Yorzoi, in cross-validation and in leave-one-genome-out evaluations, demonstrating generalization across exogenous contexts. Through an ablation analysis, we also show that Shorkie’s pretraining on fungal and native yeast sequences improves ExoShorkie’s prediction performance on exogenous DNA. Furthermore, our interpretability analyses reveal that ExoShorkie recovers canonical yeast regulatory motifs in exogenous DNA. Notably, many of these motifs are missed or predicted with opposite effects by models trained on native genomic DNA. Overall, our results suggest that regulatory rules learned from exogenous sequences are generalizable and extend beyond those inferred from the native genome. We expect ExoShorkie to be applied to design synthetic sequences with desired expression levels for various applications.

## 2 Methods

### 2.1 RNA-seq datasets

We utilized publicly available datasets that measured strand-resolved RNA-seq coverage of exogenous DNA in *Saccharomyces cerevisiae* (*S. cerevisiae*). We also utilized a strand-resolved RNA-seq dataset of the wild-type *S. cerevisiae* genome from Wery *et al.* (2023) as a reference. A summary of all datasets is provided in Table 1.

**Table 1:** Summary of RNA-seq datasets used for training and evaluation in this study.

Study	Sequence origin	Context	Length (kb)
Wery *et al.* (2023)	*S. cerevisiae*	Native	12 157
Luthra *et al.* (2024)	*H. sapiens* chr. 7	Exogenous	1571
Meneu *et al.* (2025)	*M. pneumoniae*	Exogenous	818
	*M. mycoides*		1222
Zhou *et al.* (2022)	Data storage chr.	Exogenous	255
Camellato *et al.* (2024)	*H. sapiens* hypoxanthine phosphoribosyltransferase 1 reversed (HPRT1R)	Exogenous	114
	*H. sapiens* hypoxanthine phosphoribosyltransferase 1 (HPRT1)		114

### 2.2 RNA-seq samples pre-processing

Our RNA-seq processing pipeline was adapted from the workflow described by Luthra *et al.* (2024). We applied a uniform computational pipeline to all RNA-seq datasets listed in Table 1. We performed initial quality control and adapter removal using Trimmomatic (v0.36) (Bolger *et al.* 2014). Specifically, we utilized the ILLUMINACLIP module with the TruSeq3-PE-2 adapter library (parameters: 2:30:10:2: true) and a 4 bp sliding window to trim bases where the average Phred quality fell below 10. Reads shorter than 36 bp following trimming were discarded. Post-processing sequence quality was verified using FastQC (v0.11.8) (Andrews 2010; https://www.bioinformatics.babraham.ac.uk/projects/fastqc/).

For the native yeast experiments, we used the yeast S288C reference genome (R64) for read alignment. For the exogenous experiments, we constructed a hybrid reference genome by appending the corresponding exogenous sequence to the yeast S288C (R64) reference. This hybrid reference reflects the mixed genomic content of the cells and prevents spurious alignment of yeast RNA reads to the exogenous sequence.

We conducted sequence alignment using STAR (v2.7.10a) (Dobin *et al.* 2013). We indexed the hybrid reference genomes using the parameter —genomeSAindexNbases10. We performed alignments using —twopassModeBasic, —alignIntronMax5000, and —outFilterType BySJout. We generated output files as coordinate-sorted BAM files via the —outSAMtypeBAM SortedByCoordinate parameter. For datasets with multiple replicates, we consolidated BAM files using the Picard tools (v2.18.7) (Broad Institute) MergeSamFiles utility and indexed them using Samtools (Li *et al.* 2009).

To generate the RNA-seq tracks, we utilized the bamCoverage module from deepTools (v3.1.3) (Ramírez *et al.* 2016) to produce strand-specific BigWig files at 1 bp resolution. We normalized the signal using counts per million (CPM) via the —normalizeUsingCPM option. We generated forward- and reverse-strand tracks separately using the —filterRNAstrand flag. To mitigate coverage artifacts arising from high-copy-number repeats, we excluded the rRNA locus on chromosome XII (coordinates chrXII: 369 803–499 262) during BigWig generation using the —blackListFileName parameter.

### 2.3 ExoShorkie data pre-processing

For each dataset, we segmented the base-resolution RNA-seq coverage tracks into overlapping windows according to the input length of Shorkie: 16 384 bp. We one-hot encoded the resulting windows to fit the expected input of Shorkie. We concatenated this encoding with the species one-hot vector used by Shorkie, a 165-dimensional representation in which the *S. cerevisiae* channel (index 109) is set to 1. To generate the target labels, we followed the pre-processing of the Shorkie study: we cropped 1024 bp from both ends of the window to mitigate edge effects, retaining the central 14 336 bp. The ground-truth RNA-seq signal was then summed across non-overlapping 16 bp bins, yielding a final target vector of 896 elements per window, consistent with the model’s output dimension.

To ensure the same training set size across all exogenous datasets, despite their varying total lengths, we computed a dataset-specific stride based on the training sequence length *L* and window size *W*, such that slightly more than 20 000 training windows were generated per dataset after including reverse-complement windows:


$$\text{stride}=\left\lfloor \frac{L-W}{10,000}\right\rfloor$$

To normalize the target labels during training, we applied a log transformation, log(x+1), to all 896-dimensional target vectors. Subsequently, we performed z-score normalization using a global mean and standard deviation computed across all bins and all windows in the training set. For evaluation, we used the target labels at their original coverage scale, as performance was measured using Spearman correlation, which is invariant to monotonic transformations.

### 2.4 ExoShorkie model architecture

**Figure 1:**
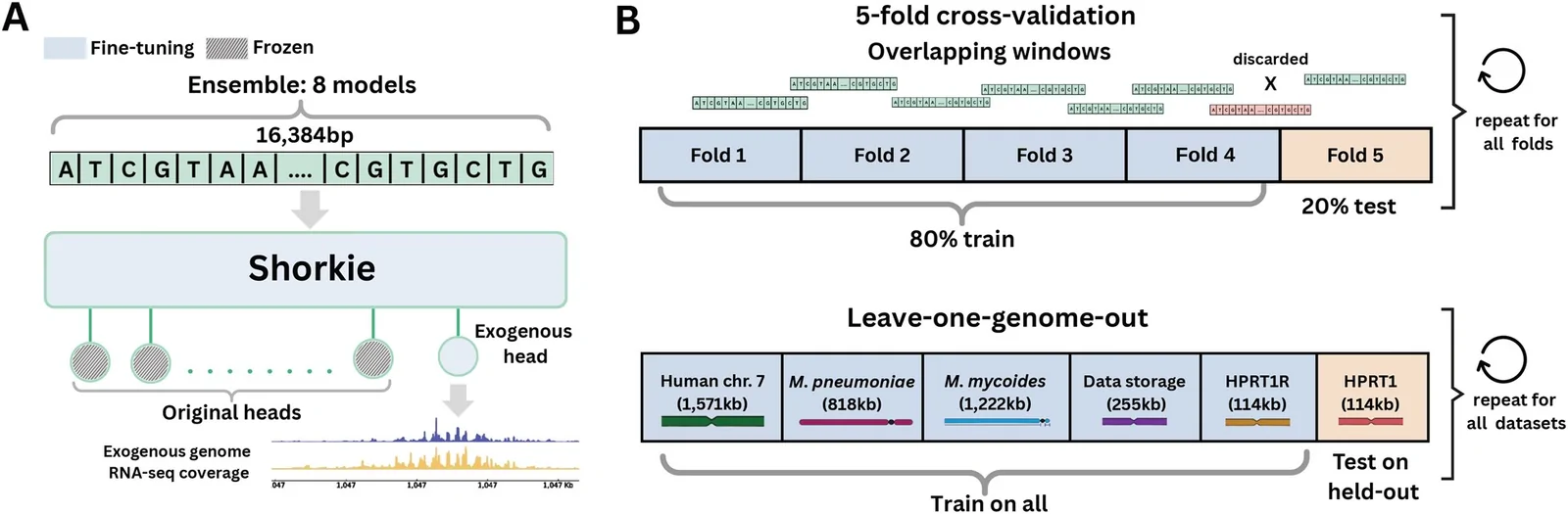
Overview of the ExoShorkie model, its fine-tuning and performance evaluation. (A) Model fine-tuning scheme. An additional prediction head is added to Shorkie, and both the trunk and the exogenous head are fine-tuned jointly to predict RNA-seq coverage from 16 384 bp-long exogenous DNA sequences expressed in yeast cells, while the original Shorkie prediction heads are frozen. To improve robustness, an ensemble of eight models is trained for each cross-validation split. (B) Evaluation strategies. In 5-fold cross-validation, we partitioned each exogenous genome by genomic coordinates into five folds, with one fold used for testing and four for training. We segmented the sequences into overlapping windows and discarded any windows that overlapped both training and test folds. In addition, we performed a leave-one-genome-out evaluation, in which we held out an entire exogenous genome to assess generalization to unseen exogenous DNA.

The Shorkie architecture is a U-Net–transformer–based model that operates on 16 384 bp input sequences (Chao *et al.* 2025). Briefly, the input sequence is first processed by a convolutional encoder with progressive downsampling, followed by a stack of transformer layers that capture long-range dependencies. A decoder with U-Net–style skip connections then restores sequence resolution and produces predictions at 16 bp resolution. We adapted this architecture to create ExoShorkie by retaining the trained Shorkie feature-extraction trunk as a backbone and adding a new task-specific dense output layer to predict the experimental tracks used in this study, while ignoring the outputs of the original Shorkie prediction heads (Fig. 1A).

### 2.5 ExoShorkie training strategy and hyper-parameters

To fine-tune Shorkie, we treated each exogenous dataset as a separate learning task and fine-tuned Shorkie independently on each dataset. We utilized the trained Shorkie feature-extraction trunk, attaching a new task-specific output layer. We then fine-tuned the Shorkie trunk and the new output layer jointly, while ignoring the outputs of the original task-specific heads (Fig. 1A).

We performed fine-tuning in two stages. First, since the original Shorkie model outputs non-strand-resolved RNA-seq coverage predictions whereas our target data are strand-resolved, we performed a native-genomic adaptation phase on native yeast RNA-seq data. We fine-tuned the model on the native Wery *et al.* dataset for five epochs with a batch size of 32 and a learning rate of $2\cdot 10^{-5}$, matching the learning rate used in the original Shorkie fine-tuning. Subsequently, we fine-tuned this adapted model on the specific exogenous dataset for three epochs to limit drift from the trained representation, with a batch size of 32 and a learning rate of $1\cdot 10^{-5}$, following learning-rate settings used for Enformer fine-tuning (Ribeiro-dos-Santos and Maurano 2025). In both stages, we utilized the Adam optimizer (default parameters: $\beta_{1}=0.9,\beta_{2}=0.999$) and minimized the mean-squared-error loss. All model fine-tuning was performed on a Linux server (Intel Xeon Gold 6338 @ 2.00 GHz, 1× NVIDIA A100 80 GB GPU, 512 GB RAM). The full fine-tuning procedure completed in under 56 hours.

### 2.6 ExoShorkie ensemble learning strategy

We employed an ensemble learning strategy in ExoShorkie to improve prediction robustness, i.e., minimize its variance. The original Shorkie model is distributed as an ensemble of eight distinct model instances, each originally trained on a different split of an 8-fold cross-validation scheme. During the native-genomic adaptation phase, we fine-tuned each of the eight Shorkie models independently, using distinct random seeds for data shuffling and initialization of the task-specific output layers. These adapted models served as the starting point for all subsequent exogenous fine-tuning. During the 5-fold cross-validation on each exogenous dataset, all eight instances were fine-tuned on the training set of each fold, resulting in a total of 40 trained ensemble models per dataset.

For cross-validation evaluation, predictions for each test sequence were obtained by averaging the outputs of the eight ensemble members trained on the corresponding folds. For leave-one-genome-out evaluation, predictions for each held-out genome were computed by averaging the outputs of all ensemble models trained on the remaining exogenous datasets.

### 2.7 Prediction performance evaluation

#### 2.7.1 Cross-validation evaluation

We evaluated prediction performance within each dataset using a 5-fold cross-validation strategy (Fig. 1B). To prevent data leakage, we partitioned each exogenous genome into five contiguous non-overlapping genomic intervals based on positional coordinates. In each iteration, four intervals (80%) were utilized for training and one interval (20%) was held out for testing. We discarded any windows spanning the train-test boundary to strictly avoid train-test overlap. Test intervals were segmented into overlapping 16 384 bp windows with a 1024 bp stride to ensure complete coverage. To verify that overlap between adjacent windows does not inflate performance estimates, we repeated the evaluation using windows with non-overlapping predicted regions. We quantified prediction performance using the median Spearman correlation coefficient between the predicted and observed coverage tracks over all test windows.

#### 2.7.2 Leave-one-genome-out evaluation

To assess the model’s ability to generalize to unseen exogenous genomes, we employed a leave-one-genome-out strategy (Fig. 1B). For this evaluation, each exogenous dataset served as a held-out test set, while predictions were generated by an ensemble comprising all models trained on the remaining datasets. The held-out genome was segmented into overlapping 16 384 bp windows with a 1024 bp stride. For each window, we averaged the predictions from all models in the ensemble to produce a final predicted RNA-seq coverage profile. We quantified prediction performance using the median Spearman correlation coefficient between the predicted and observed coverage tracks over all test windows. We compared ExoShorkie’s performance to the competing method Yorzoi (Supplementary Section S1).

### 2.8 Interpretability analysis

We interrogated the regulatory logic learned by ExoShorkie through both local interpretability via *in silico* mutagenesis (ISM) and global interpretability via F-MoDA (Yaish and Orenstein 2025) for motif finding in attribution maps (Supplementary Section S2).

## 3 Results

### 3.1 ExoShorkie achieves high predictive performance in cross-validation

We first established the baseline performance of models trained exclusively on native genomic sequences when evaluated on out-of-distribution exogenous DNA (Supplementary Section S3). We refer to this native-genome-trained baseline as *NatShorkie*. We next tested whether fine-tuning NatShorkie on exogenous DNA sequences, which we term ExoShorkie, could improve its performance on held-out sequences from the same exogenous source. We applied a 5-fold cross-validation strategy, partitioning each exogenous sequence into five parts based on genomic coordinates to avoid train-test overlaps.

**Figure 2:**
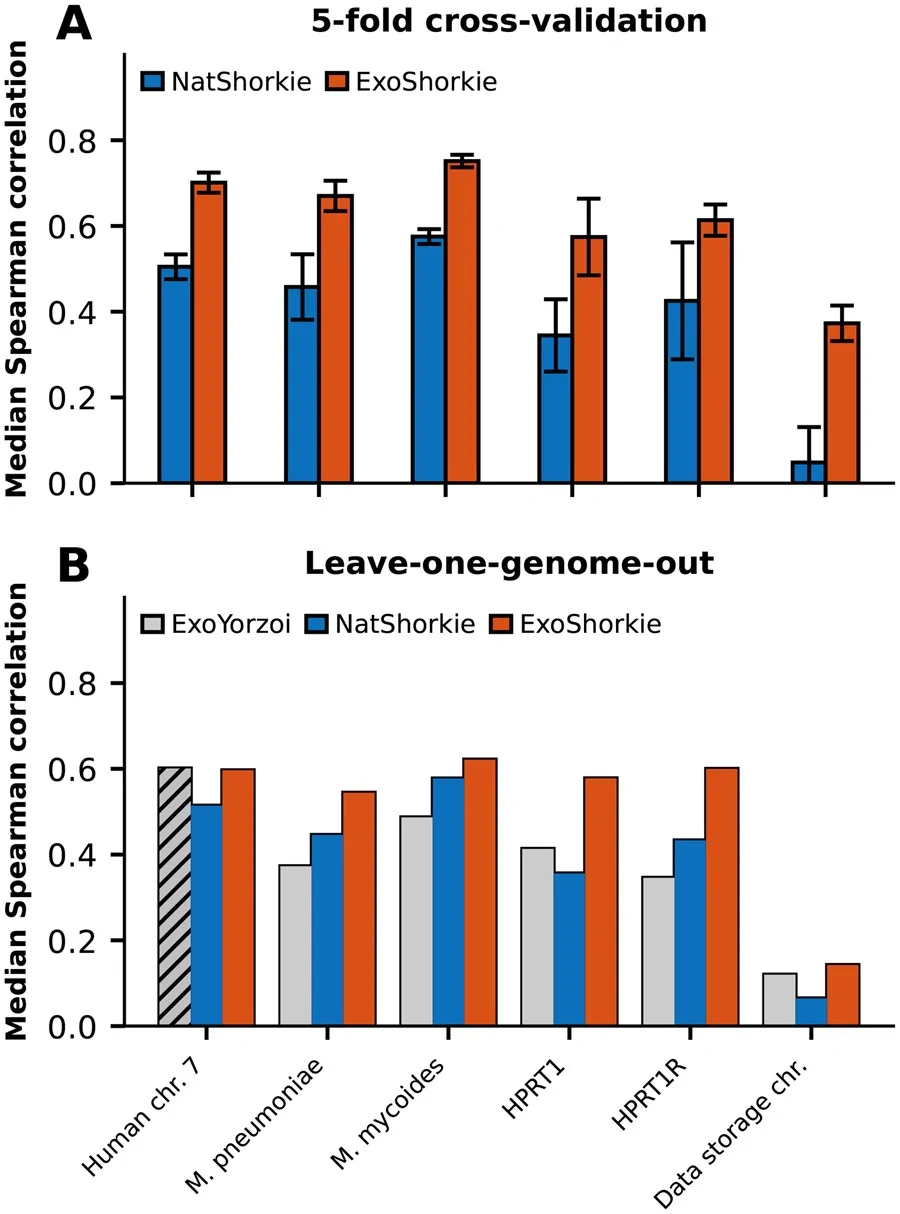
Prediction performance on exogenous genomes. (A) Comparison of median Spearman correlation over test windows between the native-genome-trained NatShorkie and ExoShorkie in 5-fold cross-validation. Error bars indicate the standard deviation across the five cross-validation test folds. (B) Comparison of median Spearman correlation over test windows between ExoYorzoi, NatShorkie, and ExoShorkie. Yorzoi was partially trained on human chromosome 7 (striped bar), whereas ExoShorkie was not exposed to this genome.

Cross-validation significantly improved performance on all datasets compared to NatShorkie, with the average median Spearman correlation across the five folds increasing by 0.17–0.32 (Fig. 2A, one-sided Wilcoxon signed-rank test on per-genome averages of per-fold median Spearman correlations, p=0.016). Performance remained high across different test-window overlap configurations (Supplementary Section S5). The highest increase was observed in the data storage chromosome, where the median Spearman correlation improved from 0.05 ± 0.08 in the native baseline to 0.37 ± 0.04. This demonstrates that ExoShorkie was able to learn regulatory rules where the native-genome-trained baseline NatShorkie failed, despite the sequence lacking evolutionary history.

### 3.2 ExoShorkie generalizes to unseen exogenous genomes

To evaluate ExoShorkie’s ability to generalize to exogenous genomes not observed during training, we performed a leave-one-genome-out evaluation. In each iteration, one exogenous genome was held out as a test set, while predictions were generated by averaging the outputs of all ensemble models trained on the remaining genomes. This task is fundamentally more challenging than cross-validation within a single study, as the model cannot rely on genome- or experiment-specific biases and must instead capture the intrinsic regulatory rules underlying the expression of exogenous DNA in yeast cells.

Across all held-out genomes, ExoShorkie consistently achieved higher prediction performance than the native-genome-trained baseline NatShorkie (Fig. 2B, one-sided Wilcoxon signed-rank tests on per-window Spearman correlations, all $p<10^{-13}$). These results indicate that ExoShorkie learns generalizable regulatory principles of exogenous gene expression that transfer to previously unseen genomes. Importantly, this evaluation setting closely reflects practical applications of ExoShorkie, where the goal is to predict the expression of newly introduced exogenous sequences for which no experimental measurements are available.

To quantify the contribution of training data diversity to generalization, we compared leave-one-genome-out performance of different subsets of the exogenous training data (Supplementary Section S6). Across all exogenous genomes, training on the full set of exogenous genomes (excluding the held-out genome) yielded the best performance. Furthermore, ExoShorkie maintained improved performance in a leave-one-study-out setting, where all data from the held-out genome’s study were excluded from training, while showing only marginal decreases in performance and even slight improvements in some cases when tested on held-out native-genome measurements (Supplementary Fig. S5).

### 3.3 ExoShorkie demonstrates improved generalization compared to ExoYorzoi

We next compared ExoShorkie’s performance on unseen exogenous genomes to ExoYorzoi, the aggregated exogenous prediction derived from Yorzoi (Schneider *et al.* 2025) (Supplementary Section S1). To enable a fair comparison, we evaluated ExoShorkie under a leave-one-genome-out setting and compared its performance to ExoYorzoi on held-out exogenous genomes.

Across all held-out genomes, ExoShorkie achieved higher or comparable median Spearman correlations relative to ExoYorzoi (Fig. 2B). ExoShorkie significantly outperformed ExoYorzoi on four out of six held-out genomes (one-sided Wilcoxon signed-rank tests on per-window Spearman correlations, all $p<10^{-27}$). On human chromosome 7, both models showed comparable performance, with ExoYorzoi achieving a slightly higher correlation. However, this genome was partially observed during ExoYorzoi’s training, whereas it was completely unseen by ExoShorkie in this evaluation. On the data storage chromosome, both models showed low performance, with only marginal differences between them and slightly higher correlations for ExoShorkie. This likely reflects the synthetic nature of the sequence, which lacks evolutionary constraints and was not shaped by natural selection.

Importantly, aside from human chromosome 7, all comparisons were performed on genomes that were unseen by both models, and therefore provide a direct evaluation of generalization to novel exogenous DNA. ExoShorkie demonstrates stronger generalization to new genomes than ExoYorzoi, consistent with its training on a more diverse set of exogenous genomes, whereas ExoYorzoi was trained exclusively on exogenous human DNA expressed in yeast.

### 3.4 Pretraining outperforms random initialization

To assess the contribution of pretraining to ExoShorkie’s prediction performance, we compared ExoShorkie models initialized by the original Shorkie model weights after RNA-seq fine-tuning on the yeast genome, with models of the same architecture initialized with random weights. We evaluated both models using the 5-fold cross-validation setting.

Across all exogenous genomes, initialization by Shorkie’s trained weights achieved higher prediction performance compared to random initialization (Supplementary Fig. S6, one-sided Wilcoxon signed-rank test on per-genome averages of per-fold median Spearman correlations, p=0.016). These results demonstrate that pretraining on native genomic sequences provides a substantial benefit for ExoShorkie’s downstream prediction performance, consistent with the improvements reported for Shorkie in the task of RNA-seq coverage prediction on the native genome in the original study.

### 3.5 Attribution maps of ExoShorkie recover known yeast TF motifs in exogenous DNA

To investigate the regulatory rules learned by ExoShorkie in exogenous sequences, we performed ISM analysis on both NatShorkie and ExoShorkie. For each exogenous genome, we computed attribution maps by performing single-nucleotide substitutions at every genomic position and measuring their effects on RNA-seq coverage predictions. We used these attribution maps for local interpretability by identifying short genomic regions with high importance for model predictions, referred to as *seqlets*, which we subsequently matched against a yeast transcription-factor (TF) motif database. In addition, we applied the global motif discovery method F-MoDA to the same ISM attribution maps (Supplementary Fig. S7). Using both local and global interpretability analyses, ExoShorkie recovered canonical yeast regulatory motifs, including Reb1, Abf1, Ume6, and the TATA-box motif, within exogenous DNA, indicating that its predictions are driven by native yeast regulatory logic acting on non-native sequences.

To focus on regions where NatShorkie and ExoShorkie disagreed in their learned regulatory logic, we computed the Pearson correlation between their contribution scores over the same windows (Supplementary Fig. S8). This window-based analysis allowed us to identify local regions with low agreement, highlighting candidate regulatory elements that are interpreted differently by the two models.

**Figure 3:**
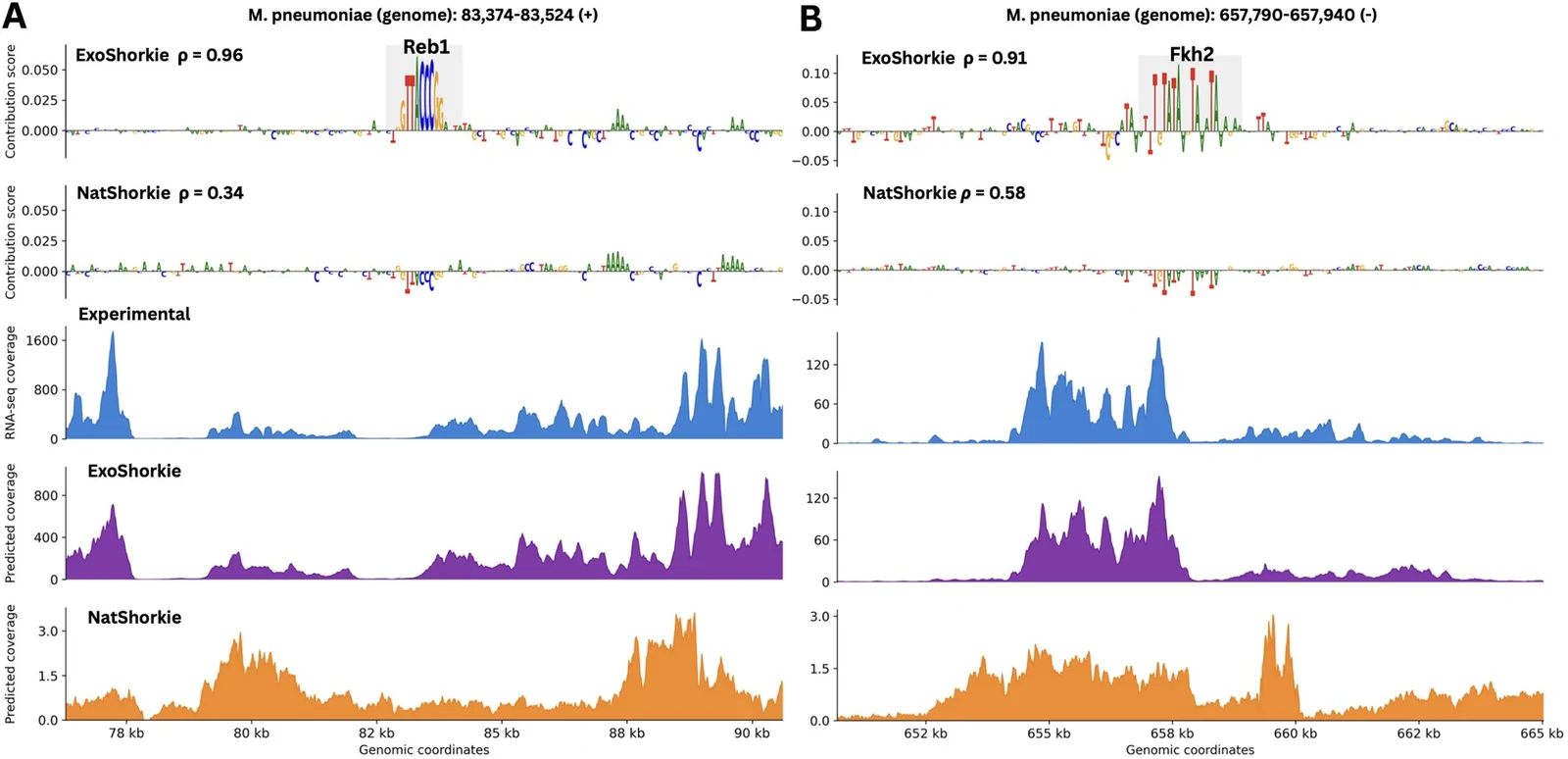
ExoShorkie recovers canonical yeast TF motifs from ISM attribution maps on the *M. pneumoniae* exogenous genome that are not recovered by NatShorkie. (A) Contribution scores from ExoShorkie (top) recover a Reb1 motif that is absent in NatShorkie (bottom). The Spearman correlation (ρ) between predicted and measured RNA-seq coverage within windows centered on the motif is significantly higher for the ExoShorkie model. ExoShorkie’s predicted coverage profile is more concordant with the true RNA-seq signal across the central 14 336 bp predicted region of the 16 384 bp window centered on the motif. (B) Contribution scores from ExoShorkie (top) recover a Forkhead (Fkh2) motif that is missed by NatShorkie (bottom). The Spearman correlation between predicted and measured coverage in motif-centered windows is significantly higher for ExoShorkie, and the predicted coverage profile is also more concordant with the true RNA-seq signal. Prediction profiles are on a normalized scale derived from training-set statistics. NatShorkie predicts lower expression magnitudes than the experimental signal due to regression toward the typical expression levels observed during NatShorkie training on endogenous yeast sequences. In contrast, ExoShorkie correctly predicts strong expression at these exogenous loci.

We then examined windows in which the Pearson correlation between contribution scores from ExoShorkie and NatShorkie was negative. Within these disagreement regions, we observed canonical yeast TF motifs that were recovered by ExoShorkie but missed by NatShorkie (Fig. 3). For windows containing these motifs, we computed the Spearman correlation between predicted and measured RNA-seq coverage and observed substantially higher correlations for ExoShorkie compared to NatShorkie. We applied the same interpretability pipeline across all exogenous genomes and similarly observed that ExoShorkie recovered exogenous-specific elements that NatShorkie missed (Supplementary Figs. S9–S13).

Together, these results demonstrate that ExoShorkie learned regulatory logic that differs from NatShorkie when modeling exogenous DNA and that this difference in regulatory rules translates into improved predictive performance.

## 4 Discussion

In this study, we demonstrated through ExoShorkie that combining transfer learning over the native yeast genome with fine-tuning on diverse exogenous RNA-seq datasets substantially improves performance on exogenous RNA-seq coverage prediction. By leveraging Shorkie, a pretrained state-of-the-art yeast expression model, and fine-tuning it on multiple recently published RNA-seq datasets from exogenous genomes, ExoShorkie achieves state-of-the-art performance in predicting the expression of held-out exogenous DNA sequences, outperforming both a native-genome-trained baseline and Yorzoi, which is the only existing exogenous RNA-seq coverage prediction method. ExoShorkie employs an efficient fine-tuning strategy that leverages pretrained regulatory representations by introducing a lightweight prediction head. This design reduces computational requirements, enabling ensemble learning that further improves robustness and reduces prediction variance. Our results demonstrate that training on diverse exogenous genomes from multiple sources substantially improves generalization to new exogenous genomes. Overall, transfer learning from a yeast expression model consistently improves prediction performance across all evaluated exogenous genomes.

Our interpretability analysis via ISM shows that ExoShorkie captures canonical yeast TF motifs, including Reb1, Abf1, Ume6, and the TATA-box, in exogenous sequences, despite the fact that these sequences were not constrained by yeast evolution. Moreover, comparison of ISM attribution maps in regions where ExoShorkie and the native-genome-trained baseline NatShorkie disagree revealed distinct regulatory motifs identified by ExoShorkie and missed by NatShorkie (Fig. 3). These regions correspond to windows in which ExoShorkie achieves substantially higher concordance with the ground-truth RNA-seq profile compared to NatShorkie, suggesting that the distinct regulatory logic learned by ExoShorkie is the reason for its improved prediction performance.

There are several limitations to our study. First, our analysis is constrained by the small number of studies that have generated RNA-seq data of exogenous genomes in yeast. Despite recent advances in synthetic genomics, the synthesis and delivery of exogenous DNA remain labor- and resource-intensive, which restricts the availability of large and diverse datasets. We expect that future technological developments will help expand the scale of exogenous RNA-seq data that can be used to train ExoShorkie. Second, our study is currently limited to a yeast host system. While yeast is a widely used model organism for studying gene regulation, the regulatory rules learned by ExoShorkie may be yeast-specific and may not directly generalize to other host organisms. Third, ExoShorkie achieves its lowest performance on highly artificial data storage DNA. Sequences that lack known evolutionary or designed regulatory elements may represent a more challenging prediction task and require substantially larger and more diverse training datasets than are currently available.

There are several directions in which we plan to extend ExoShorkie in the future. First, experimental validation of ExoShorkie’s model-identified regulatory elements would strengthen the evidence for ExoShorkie’s ability to recover exogenous-specific elements. Second, we plan to extend the model to jointly predict multiple genomic and epigenomic tracks by incorporating datasets from additional assays, such as ATAC-seq and ChIP-seq, measured on native and/or exogenous genomes. Following such prediction, we plan to run annotation methods to propose novel promoters and enhancers over exogenous genomes and then to focus our interpretability analysis on such regions to better understand their regulatory logic. As more exogenous datasets become available, we also aim to systematically investigate the factors driving cross-genome generalization and the differences in regulatory grammar between native and exogenous sequences. Finally, we plan to generalize our approach to exogenous RNA-seq experiments in mammalian systems (Brosh *et al.* 2023), in combination with recent mammalian sequence-to-expression models (Linder *et al.* 2025, Avsec *et al.* 2026).

## 5 Conclusion

We introduced ExoShorkie, an interpretable method for predicting RNA-seq coverage from exogenous DNA sequences that generalizes across unseen genomes and outperforms existing methods. Our results show that ExoShorkie learns regulatory patterns that differ between native and exogenous sequence contexts. Beyond prediction, ExoShorkie provides a foundation for the rational design of long multi-gene synthetic DNA constructs with predictable expression levels by sequence optimization methods. In addition, ExoShorkie offers a general framework for studying the transcriptional regulation of exogenous sequences in yeast, advancing our understanding of gene regulation beyond native genomic contexts.

## Author contributions

Jonathan Mandl (Data curation, Formal analysis, Investigation, Methodology, Software, Validation, Visualization, Writing—original draft), and Yaron Orenstein (Conceptualization, Funding acquisition, Project administration, Resources, Supervision, Writing—review & editing)

## Supplementary Material

btag369_Supplementary_Data

## Data Availability

The source code for ExoShorkie is available at https://github.com/OrensteinLab/ExoShorkie. An archived release of the software is available on Zenodo at https://doi.org/10.5281/zenodo.20628032. The reference exogenous genome sequences and processed RNA-seq datasets used in this study are available on Figshare at https://doi.org/10.6084/m9.figshare.31075375. Trained ExoShorkie models are available on Hugging Face at https://huggingface.co/Jonathan-Mandl/ExoShorkie-models.
