# Supplementary material for: ExoShorkie: predicting RNA-seq coverage of exogenous genomes in yeast by transfer learning
Source: Bioinformatics. 2026 Aug 21;42(Suppl 2):btag369. doi: 10.1093/bioinformatics/btag369 (PMC13501306; doi:10.1093/bioinformatics/btag369)
Supplement: Supplementary file 1 [file btag369_supplementary_data.pdf]

Supplementary information for: ExoShorkie: Predicting RNA-seq coverage of exogenous genomes in yeast by transfer learning

S1. Comparison to Yorzoi

To enable a fair comparison between ExoShorkie and Yorzoi, the only current method for RNA-seq coverage prediction of exogenous genomes in yeast, we modified Yorzoi's prediction pipeline to account for differences in input length, output resolution, and the number of predicted tracks. Yorzoi operates on input windows of 4,992 bp and predicts coverage over the central 3,000 bp at a 10 bp resolution. We first applied Yorzoi to the full test sequences using a stride of 3,000 bp, corresponding to its predicted region, to generate genome-wide predictions for all 162 output tracks.

We then unbinned these predictions and inverse-transformed them using Yorzoi's built-in utilities to recover coverage values at single-nucleotide resolution. We subsequently averaged Yorzoi's predicted single-nucleotide coverage across the 10 exogenous human RNA-seq output tracks to obtain a single aggregated prediction for exogenous DNA, which we refer to as *ExoYorzoi*. We segmented the resulting genome-wide predictions into overlapping windows matching ExoShorkie's input length using a stride of 1,024 bp. For each window, we extracted the central 14,336 bp region and summed the predicted coverage into non-overlapping 16 bp bins, yielding an 896-dimensional prediction vector. Finally, we computed the median Spearman correlation coefficient between ExoYorzoi's predictions and the corresponding experimentally measured RNA-seq coverage across all test windows.

S2. Interpretability analysis

S2.1. ExoShorkie model distillation

To enable ISM with a single model, we distilled each dataset-specific ExoShorkie ensemble into a student network trained to match the ensemble's average prediction.

For each dataset, we first constructed a base set of approximately 10,000 overlapping input sequences using our dataset-specific stride strategy. We then generated augmented training examples using a sequence-augmentation pipeline adapted from AlphaGenome [Avsec et al., 2026]. Specifically, for each sampled sequence we applied: (i) reverse-complement augmentation with probability 0.5, (ii) random point mutations affecting 4% of positions, and (iii) structural variations consisting of random insertions, deletions, and inversions. The number of structural variation events per sequence was drawn from a Poisson distribution with $\lambda = 1$, and the length of each variation was sampled uniformly from $[1, 20]$ bp.

We created 50,000 synthetic training sequences by sampling from the base set of sequences with replacement and applying the augmentation pipeline. We then obtained training targets for the student model by running the corresponding 40-model ensemble on each synthetic sequence and averaging the predicted profiles across ensemble members.

The student model used the same architecture as ExoShorkie and was initialized from the trained Shorkie fold-0 weights, with the task-specific output layer randomly initialized. We trained the student model for 20 epochs with a batch size of 64 using the Adam optimizer (default parameters, consistent with ExoShorkie training) and a learning rate of $2 \cdot 10^{-5}$. After training, the student's predictions were highly correlated with the ensemble outputs (Pearson $r > 0.98$ on the distillation set).

S2.2. In silico mutagenesis analysis of ExoShorkie

We implemented an ISM pipeline adapted from the Shorkie model [Chao et al., 2025]. Given the lack of prior regulatory annotations for the exogenous sequences, we performed systematic mutagenesis of every nucleotide across each exogenous genome. We utilized a sliding window with a stride of 14,336 bp to ensure that each position was located within the prediction region of the model at least once.

For each window, we substituted every base with the three alternative nucleotides to generate coverage predictions by the model. We inverse-transformed these raw log Z-score outputs to their original coverage scale using the training-derived mean and standard deviation, and summed the predicted coverage values across all output bins for both the mutated and reference sequences. We then defined the ISM score matrix $M_{i,n}$, where each entry corresponds to the $\log_2$ fold-change in predicted coverage obtained by substituting the reference nucleotide at genomic position i with nucleotide n:

$$M_{i,n} = \log_2\left(\sum_{j\in\mathcal{B}} \hat{y}_j^{\text{alt}} + 1\right) - \log_2\left(\sum_{j\in\mathcal{B}} \hat{y}_j^{\text{ref}} + 1\right)$$

where $\hat{y}_j^{\text{alt}}$ and $\hat{y}_j^{\text{ref}}$ denote the model-predicted coverage for genomic bin j for the mutated and reference sequences, respectively, and $\mathcal{B}$ indexes the set of output bins (896 bins) produced by the model for a given window. We next centered the attribution scores

by subtracting the mean over the four nucleotides at each position:

$$\tilde{M}_{i,n} = M_{i,n} - \frac{1}{4} \sum_{n' \in \{A,C,G,T\}} M_{i,n'}$$

To focus exclusively on reference-base contributions, we computed:

$$\mathrm{C}_i = \sum_{n \in \{A,C,G,T\}} \tilde{M}_{i,n} \cdot H_{i,n}$$

where $H_{i,n} \in \{0,1\}$ denotes the one-hot indicator of the reference nucleotide at position i. This procedure yields a vector of nucleotide-level contribution scores across the sequence, which we use for visualization and subsequent motif discovery for each exogenous genome (Fig. S1).

S2.3. Seqlet calling and motif identification

We utilized the nucleotide-level contribution scores generated by the ISM pipeline to identify potential regulatory regions within the attribution maps. To identify seqlets with high contribution scores, we applied the recursive seqlet calling algorithm from the tangermeme toolkit [Schreiber, 2025a]. We executed the seqlet-calling algorithm with a minimum seqlet length of 6 bp, a maximum seqlet length of 25 bp, and a p-value threshold of 0.001.

Following the identification of high-importance seqlets, we matched them to a motif database using the same tangermeme toolkit. The tangermeme toolkit leverages tomtom-lite [Schreiber, 2025b], an optimized implementation of the Tomtom motif similarity algorithm [Gupta et al., 2007], to calculate alignments between one-hot encoded seqlets and a database of known position weight matrices. For this analysis, we used the SwissRegulon database, which contains 158 curated yeast-specific TF motifs [Pachkov et al., 2012]. We retained seqlets that had significant matches to motifs below an E-value threshold of 0.05.

S2.4. Global motif discovery using F-MoDA

We applied F-MoDA [Yaish and Orenstein, 2025], a global motif discovery method that clusters high-contribution seqlets derived from attribution maps. As input, we used the mean-centered ISM score matrix, obtained by subtracting the average attribution across the four nucleotides at each position (Section S2.2). To enable genome-wide motif discovery, attribution maps were segmented into overlapping 2,000 bp windows with 50% overlap and provided as input to F-MoDA. We retained motif clusters with support greater than 10, where support denotes the number of contributing seqlets assigned to the cluster. We compared the resulting motifs against known TF binding motifs in the same manner as we compared the seqlets (Section S2.3).

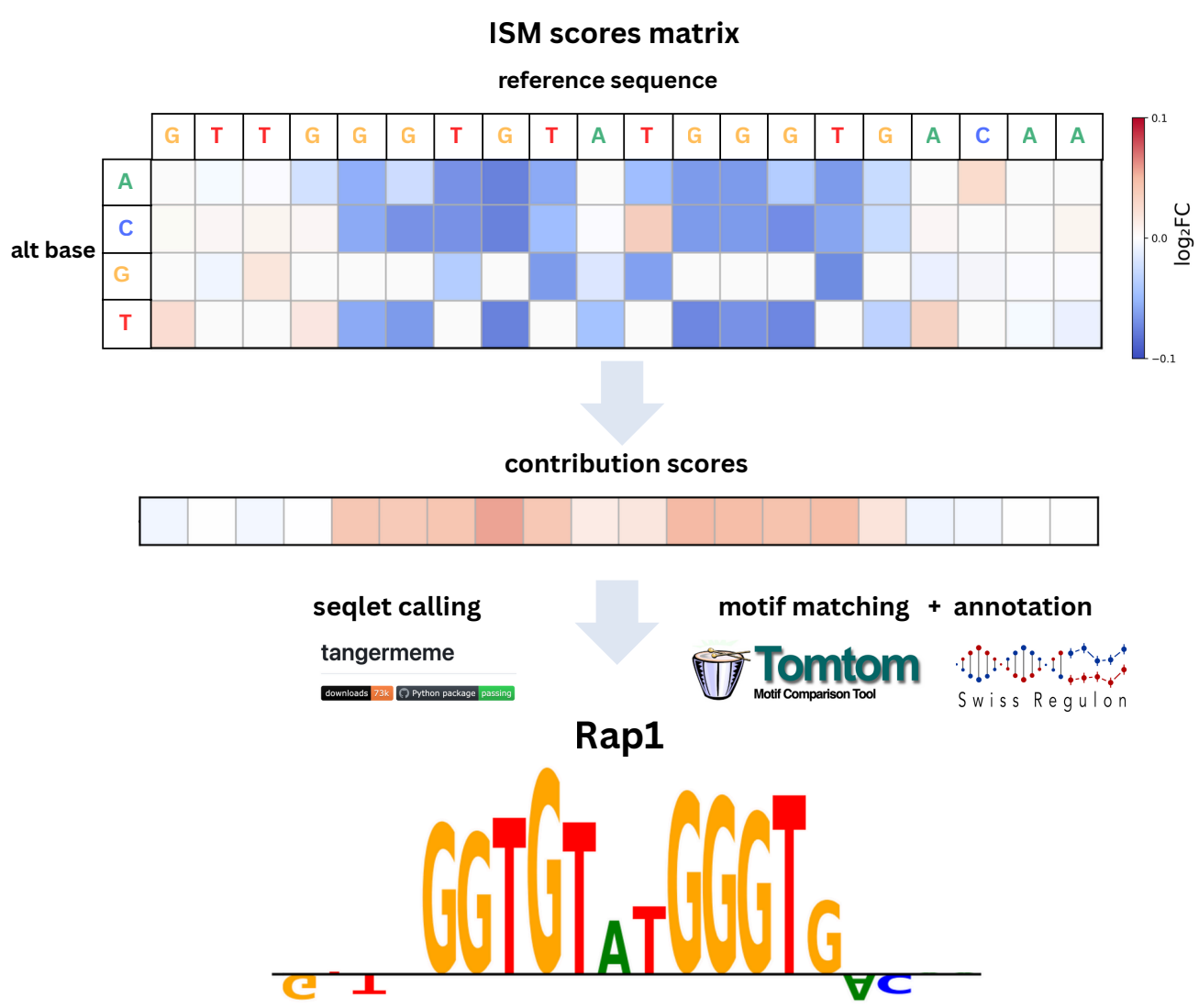

Fig. S1. ISM pipeline schematic. For each window, each nucleotide position was substituted with the three alternative bases, and an ISM score matrix was computed as the $\log_2$ fold-change ($\log_2$FC) between the summed predicted coverage of the alternative and reference sequences. At each nucleotide position, the mean $\log_2$FC across the four nucleotides was subtracted, and reference-base contribution scores were extracted. These nucleotide-level contribution scores were subsequently used for seqlet calling and motif matching against the SwissRegulon database [Pachkov et al., 2012].

S3. Native-genome-trained models achieve low prediction performance on exogenous genomes

To establish a baseline for out-of-distribution generalization, we evaluated models trained exclusively on native genomic sequences on exogenous DNA. Since we fine-tune Shorkie on exogenous data, we used the trained Shorkie model, after adapting it for strand-resolved RNA-seq coverage prediction, as our native-genome-trained baseline, which we term *NatShorkie*. We assessed NatShorkie's performance by evaluating it on the same 5-fold data splits used for ExoShorkie and averaging the per-fold median Spearman correlations.

As expected for this out-of-distribution task, the median Spearman correlations were relatively low (Fig. 2A in the main text): between 0.3 and 0.6 on evolutionary-constrained exogenous genomes, and even lower for the data storage chromosome (0.05), which was never shaped by evolutionary selection. This behavior is consistent with prior work showing that Enformer [Avsec et al., 2021], a sequence-to-function model trained on native genomic DNA, generalizes poorly when evaluated on rearranged or synthetic regulatory elements introduced into endogenous loci [Ribeiro-dos Santos and Maurano, 2025].

We hypothesized that the lower prediction performance may be partially explained by differences in sequence composition between the yeast genome and the exogenous genomes. To investigate this, we conducted a comparative analysis of sequence composition across the exogenous genomes (Section S4).

S4. Exogenous genome composition statistics

We analyzed the sequence composition of all exogenous genomes used in this study, as well as that of the *S. cerevisiae* genome. For each genome, we computed the GC content, defined as the proportion of guanine (G) and cytosine (C) nucleotides relative to the total genome length. We also calculated the CpG observed-to-expected ratio, defined as:

$$\mathrm{CpG}_{\mathrm{obs/exp}} = \frac{f_{\mathrm{CG}}}{f_{\mathrm{C}} \cdot f_{\mathrm{G}}}$$

where f_{CG} denotes the frequency of the CpG dinucleotide, and f_{C} and f_{G} denote the frequencies of cytosine and guanine nucleotides, respectively.

In addition, we performed a dinucleotide enrichment analysis by computing, for each genome, the frequency of each dinucleotide normalized by its corresponding frequency in the yeast genome. We observed substantial differences in sequence composition across the exogenous genomes, with particularly pronounced variation in the CpG observed-to-expected ratio (Fig. S2).

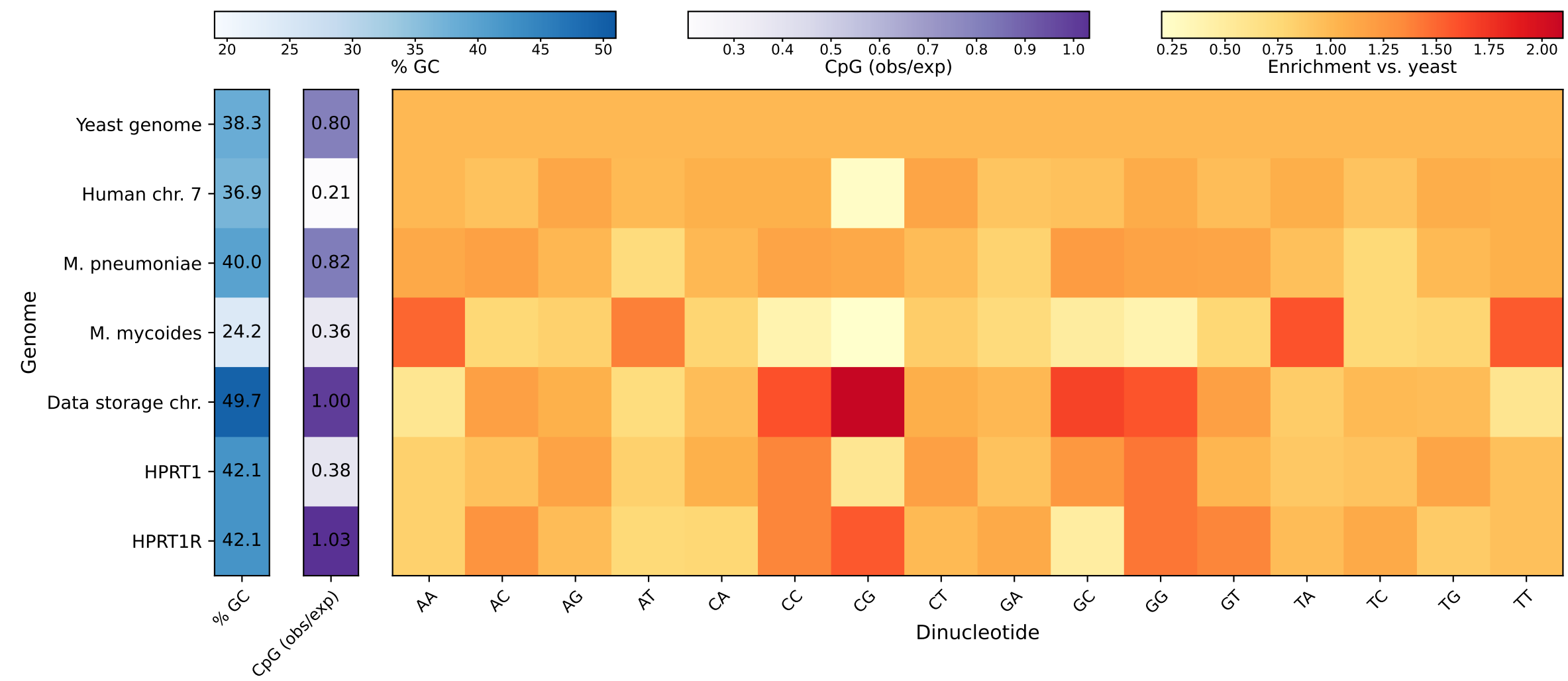

Fig. S2. Dinucleotide composition of exogenous genomes relative to the yeast genome. The central heatmap shows dinucleotide enrichment in each exogenous genome relative to the yeast genome. The left panels display overall GC content and CpG observed-to-expected ratio for each exogenous genome.

S5. Impact of test-window overlap on performance estimates

To ensure that the use of overlapping test windows (1,024 bp stride) does not bias performance estimates, we evaluated ExoShorkie under both the 5-fold cross-validation and leave-one-genome-out settings using two stride sizes: 1,024 bp and 14,336 bp (Fig. S3). The latter ensures that predicted regions do not overlap between adjacent windows.

Across both evaluation settings and all genomes, we observed only minor differences in median Spearman correlation between the two stride configurations. These results indicate that the use of overlapping windows does not inflate performance estimates and that the reported prediction accuracy reflects genuine generalization rather than redundancy introduced by window overlap.

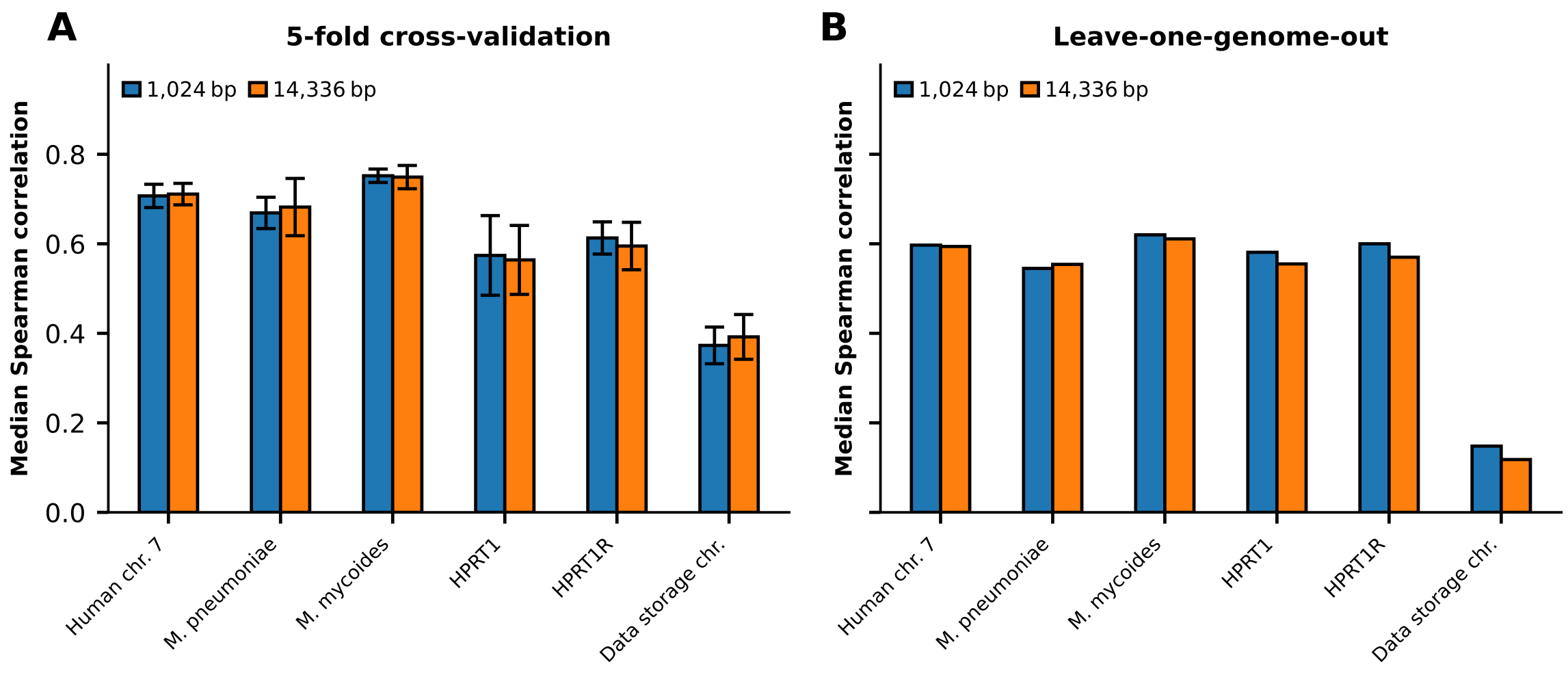

Fig. S3. Prediction performance under different test-window stride configurations. (A) Comparison of median Spearman correlation across test windows when using a 1,024 bp stride versus a 14,336 bp stride in 5-fold cross-validation. Error bars indicate the standard deviation across the five cross-validation test folds. (B) Comparison of median Spearman correlation across test windows using a 1,024 bp stride and a 14,336 bp stride in leave-one-genome-out evaluation.

S6. Leave-one-genome-out ablation analysis

To assess the contribution of the diversity of exogenous sequence data to the generalization ability of ExoShorkie, we performed an ablation analysis under the leave-one-genome-out evaluation setting. In this analysis, model performance was evaluated on each held-out exogenous genome while varying the subset of training genomes used.

Specifically, we compared four training regimes: (1) Bacteria-only, where predictions were obtained using ensembles trained on the bacterial genomes *M. pneumoniae* and *M. mycoides*. (2) Human-only, where predictions were obtained using ensembles trained on the human-derived genomes (human chromosome 7 and HPRT1). (3) Synthetic-only, where predictions were obtained using ensembles trained on the fully synthetic genomes (data storage chromosomes and HPRT1R) (4) Full training data, corresponding to the standard leave-one-genome-out setting, where predictions were obtained using ensembles trained on all available genomes except the held-out test genome.

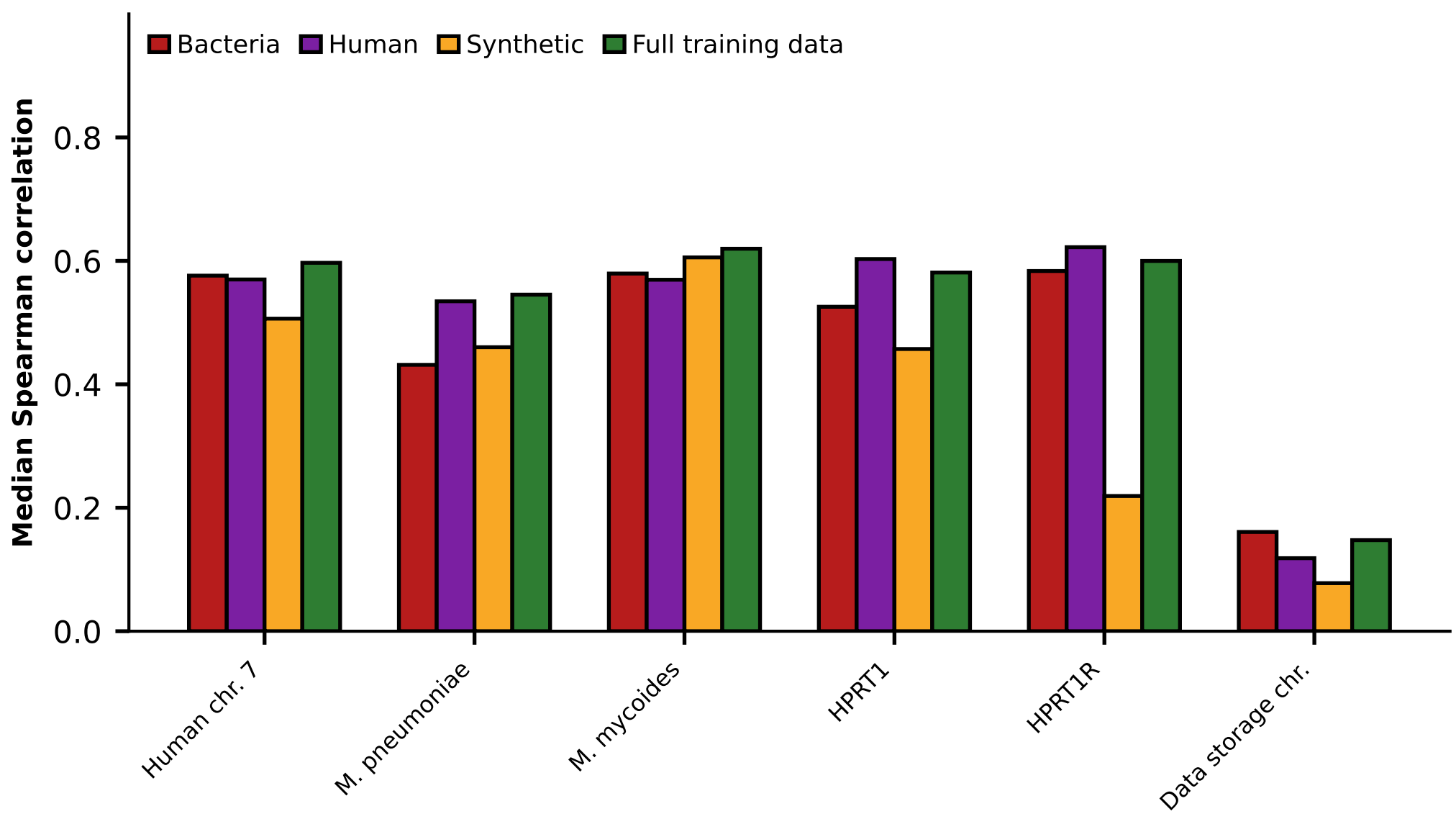

Fig. S4. Ablation study comparing model performance (median Spearman correlation) under leave-one-genome-out evaluation. Models were trained using different subsets of training genomes: only bacterial genomes (Bacteria), only human genomes (Human), only synthetic genomes (Synthetic), or all genomes (Full training data).

Across all exogenous genomes, training on the full set of available exogenous genomes (excluding the held-out test genome) resulted in the highest or near-highest performance, highlighting the importance of diverse exogenous training data for improving generalization to unseen exogenous genomes (Fig. S4).

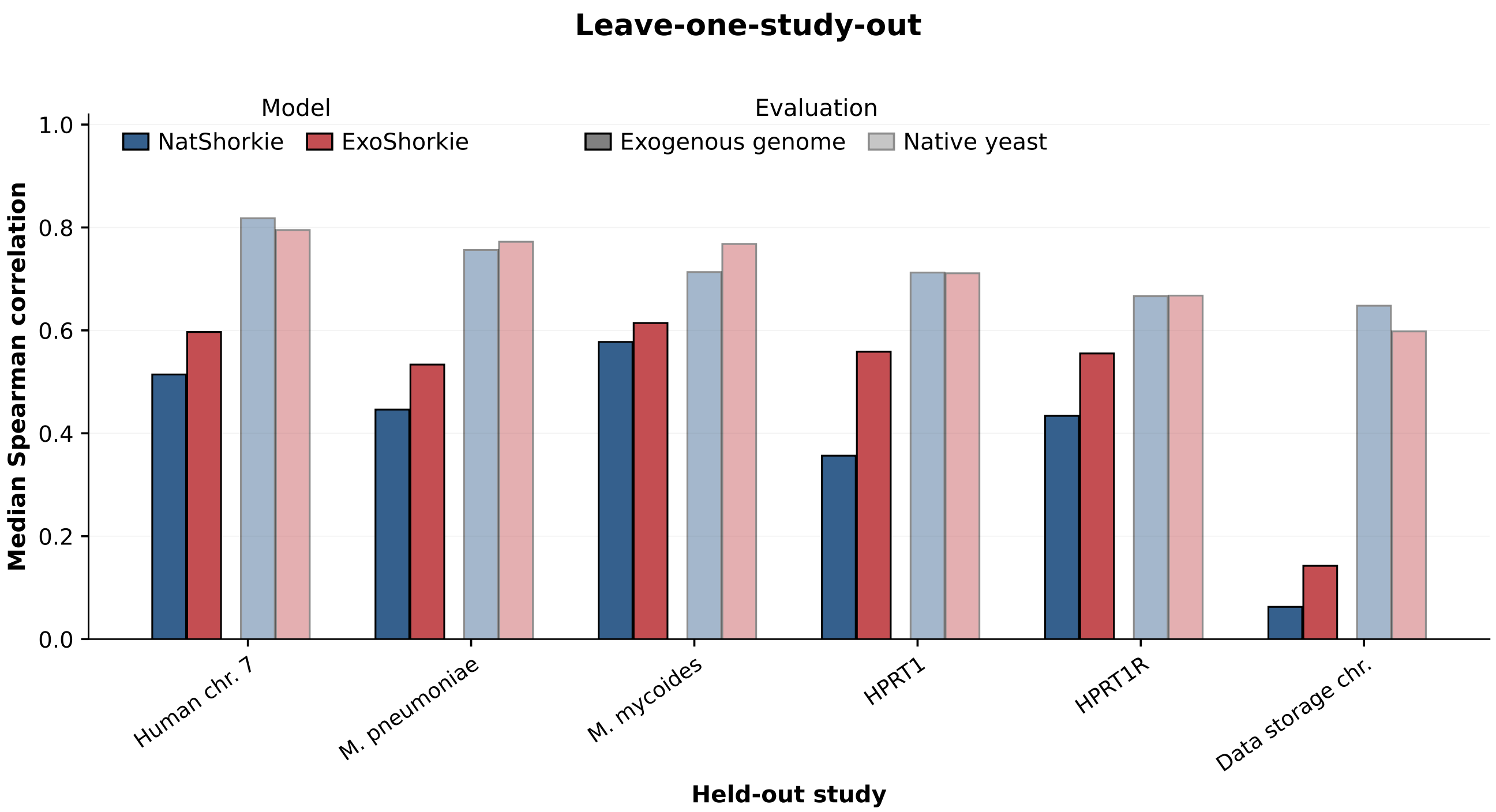

Fig. S5. Comparison of median Spearman correlation between NatShorkie and ExoShorkie in the leave-one-study-out setting. In each iteration, all genomes originating from one study were excluded from training, and predictions for sequences from each held-out genome were generated by averaging the outputs of ensemble models trained on the remaining studies. Dark bars correspond to evaluation on held-out exogenous genomes using a stride of 1,024 bp, while light bars correspond to evaluation on measurements of all native yeast chromosomes from each held-out study using a stride of 14,336 bp. ExoShorkie consistently outperformed NatShorkie on held-out exogenous genomes and largely preserved performance on native yeast sequences, with slight improvements observed in some cases.

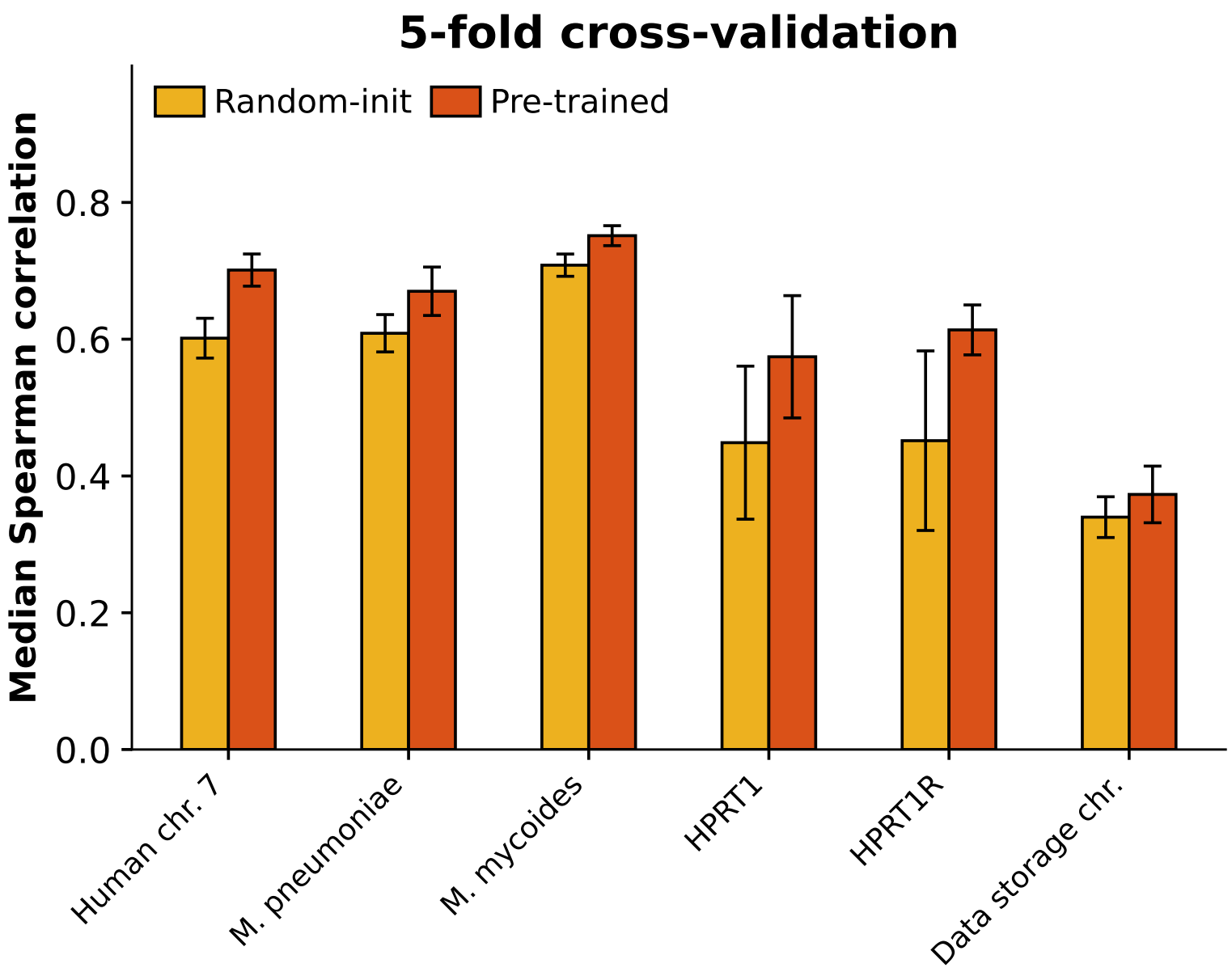

Fig. S6. Comparison of median Spearman correlation between a pretrained ExoShorkie model, initialized by the original Shorkie model weights after RNA-seq fine-tuning on the yeast genome, and a randomly initialized model (Random-init) with the same architecture, evaluated using the same 5-fold cross-validation setting. Performance is computed over test windows for each exogenous dataset. Error bars indicate the standard deviation across the five cross-validation test folds.

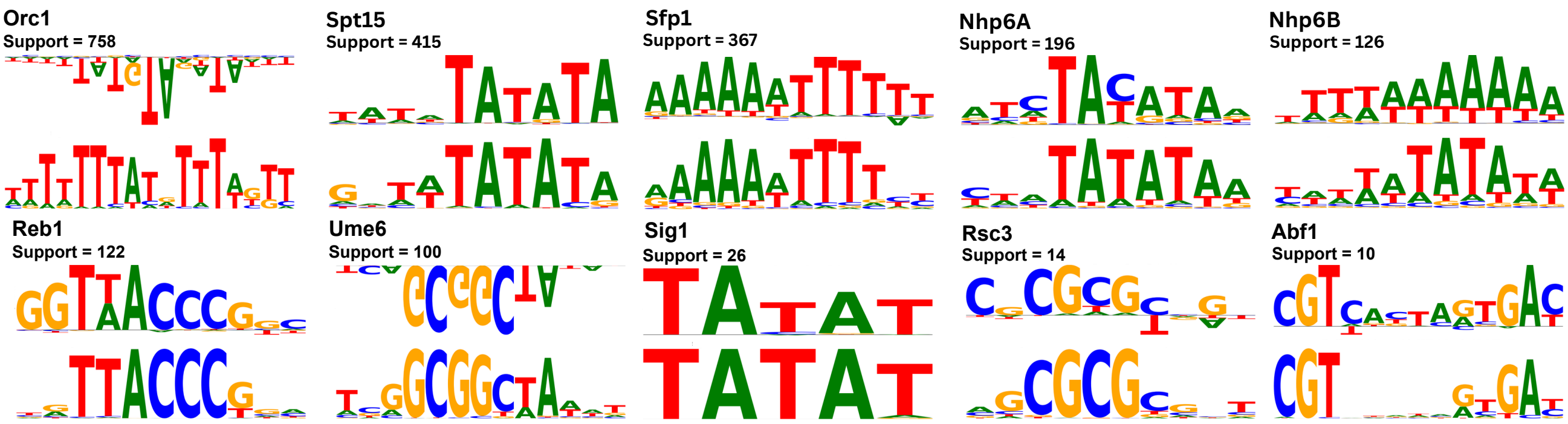

Fig. S7. Global regulatory motifs discovered by F-MoDA from genome-wide ISM attribution maps and their significant matches to known yeast TF motifs in the SwissRegulon database. For each motif pair, the top logo shows the contribution weight matrix identified by F-MoDA (cluster support shown above), and the bottom logo shows the aligned SwissRegulon motif. We report the 10 motif clusters with the highest normalized support across exogenous genomes, where normalized support is defined as the cluster support divided by the total number of motif clusters identified for the corresponding genome. Only motif clusters with support ≥ 10 and statistically significant similarity to known yeast TF motifs (Tomtom E-value < 0.05) are shown.

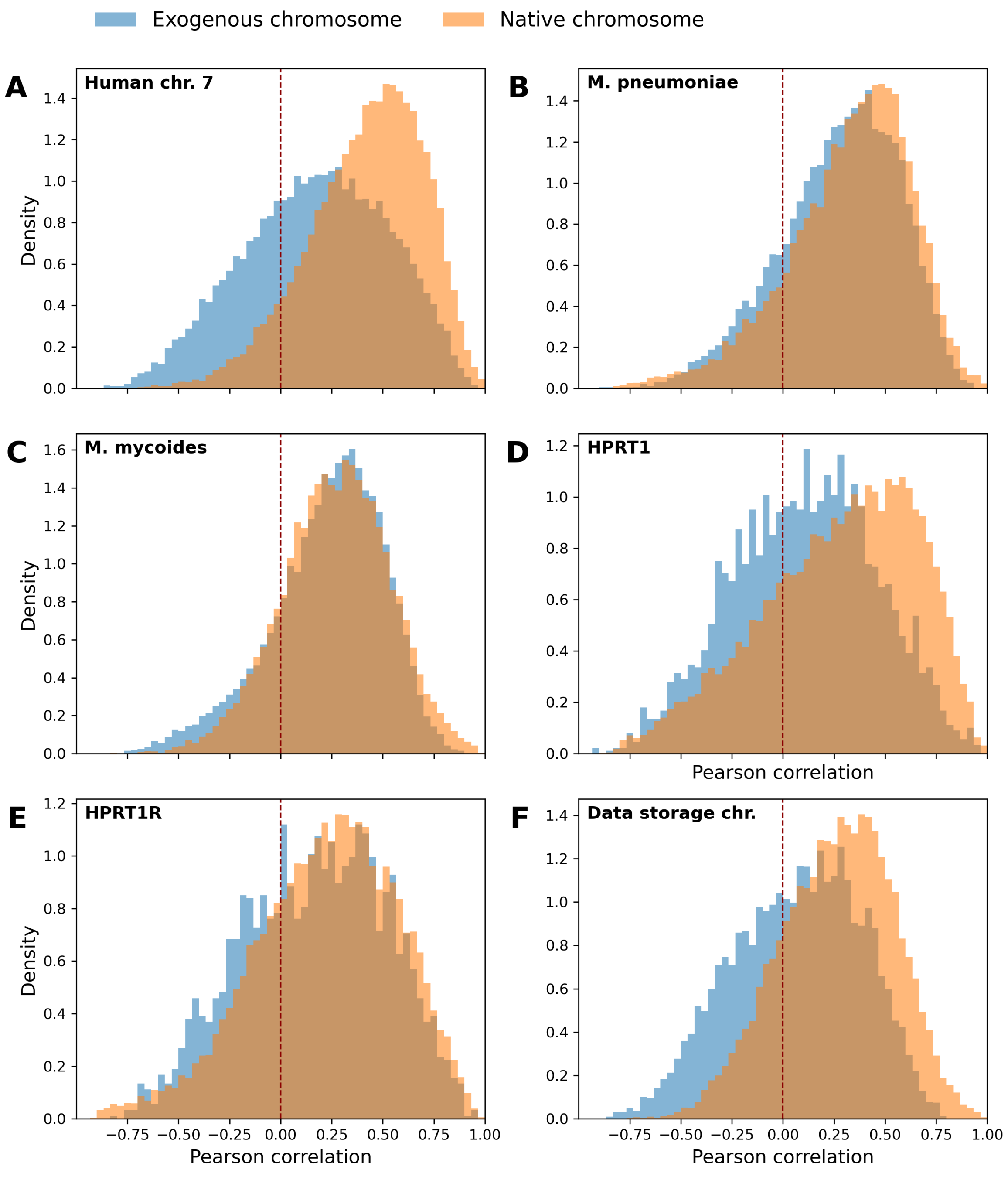

Fig. S8. Distribution of Pearson correlations between NatShorkie and ExoShorkie attribution scores across exogenous sequence windows and native yeast chromosome X windows. For each exogenous genome, the corresponding distilled ExoShorkie model was used to generate attribution scores for both the exogenous genome and yeast chromosome X, and these were compared to attribution scores generated by NatShorkie for the same sequences. Panels correspond to (A) human chromosome 7, (B) *M. pneumoniae*, (C) *M. mycoides* , (D) HPRT1 , (E) HPRT1R , and (F) data-storage chromosome. Correlations along native yeast sequences are higher since both models were trained on the native yeast genome, while only ExoShorkie was trained on exogenous genomes.

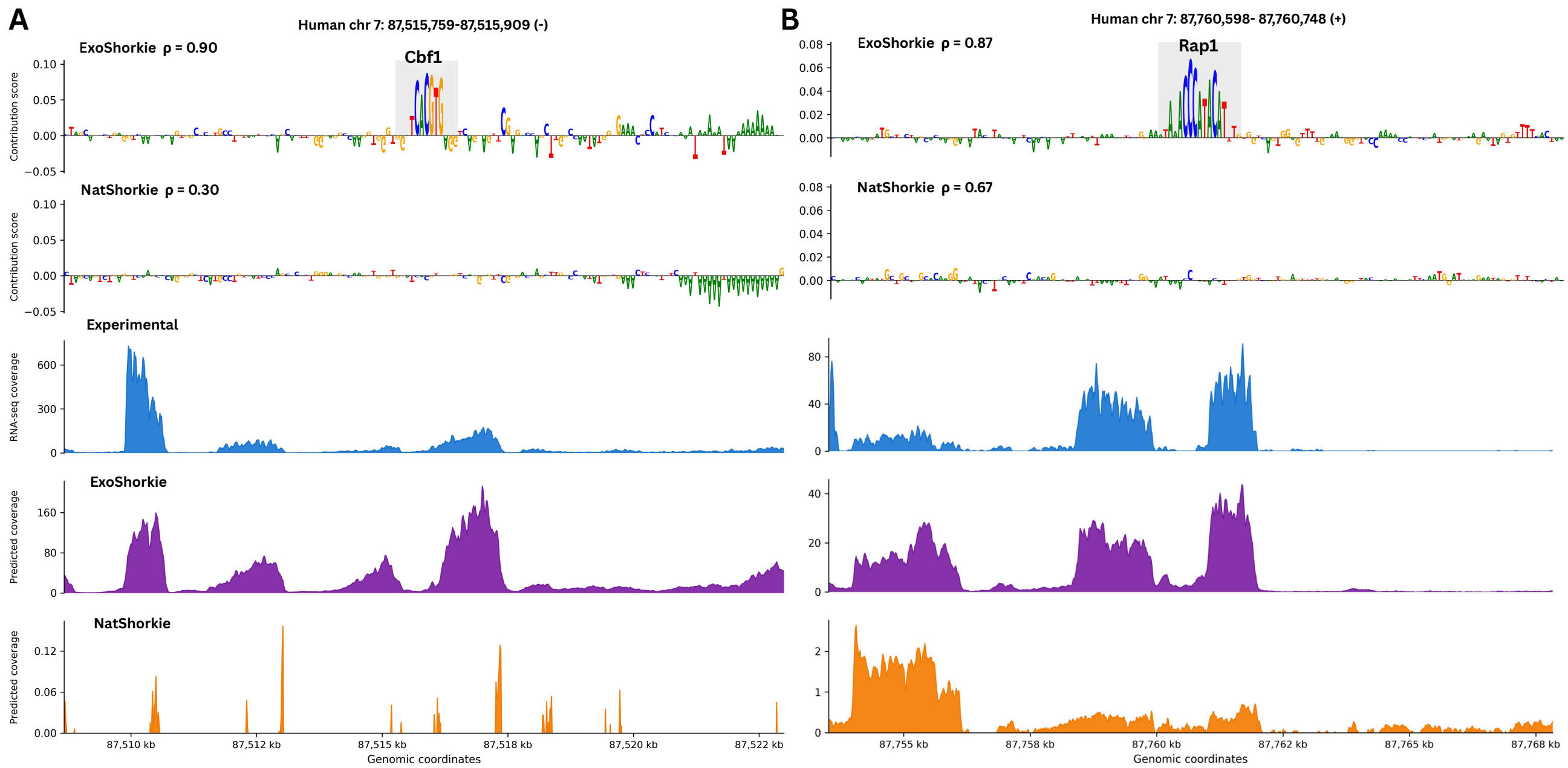

Fig. S9. ExoShorkie recovers canonical yeast TF motifs from ISM attribution maps on the exogenous human chromosome 7 that are diffuse or absent in NatShorkie. (A) Contribution scores from ExoShorkie (top) recover a Cbf1 motif that is not recovered by NatShorkie (bottom). (B) Contribution scores from ExoShorkie (top) recover a Rap1 motif that is missed by NatShorkie (bottom). For both loci, the Spearman correlation (ρ) between predicted and measured RNA-seq coverage in motif-centered windows is significantly higher for ExoShorkie. ExoShorkie's predicted coverage profile is more concordant with the true RNA-seq signal across the central 14,336 bp predicted region of the 16,384 bp window centered on each motif.

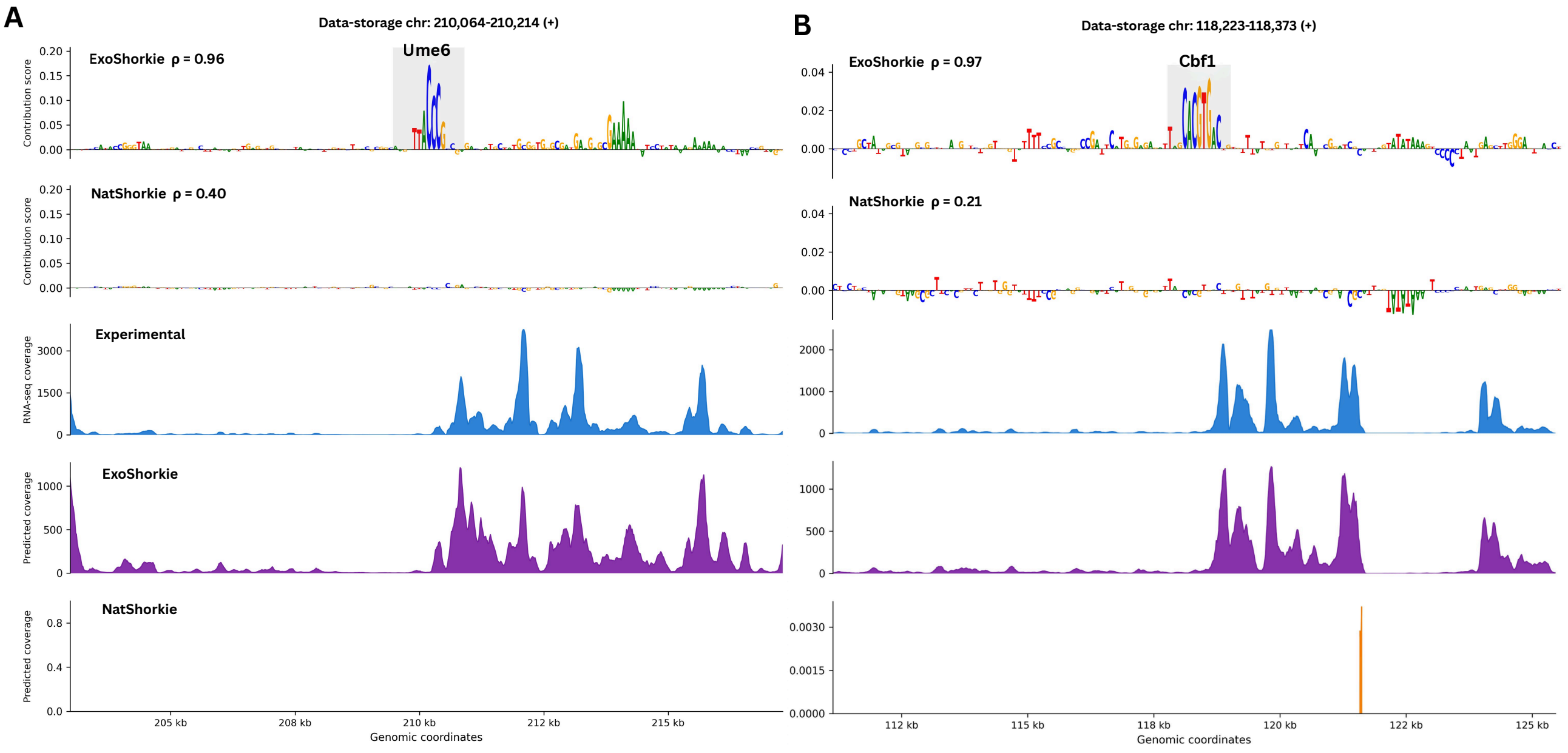

Fig. S10. ExoShorkie recovers canonical yeast TF motifs from ISM attribution maps on the exogenous data storage chromosome that are diffuse or absent in NatShorkie. (A) Contribution scores from ExoShorkie (top) recover a Ume6 motif that is not recovered by NatShorkie (bottom). (B) Contribution scores from ExoShorkie (top) recover a Cbf1 motif that is missed by NatShorkie (bottom). For both loci, the Spearman correlation (ρ) between predicted and measured RNA-seq coverage in motif-centered windows is significantly higher for ExoShorkie. ExoShorkie's predicted coverage profile is more concordant with the true RNA-seq signal across the central 14,336 bp predicted region of the 16,384 bp window centered on each motif.

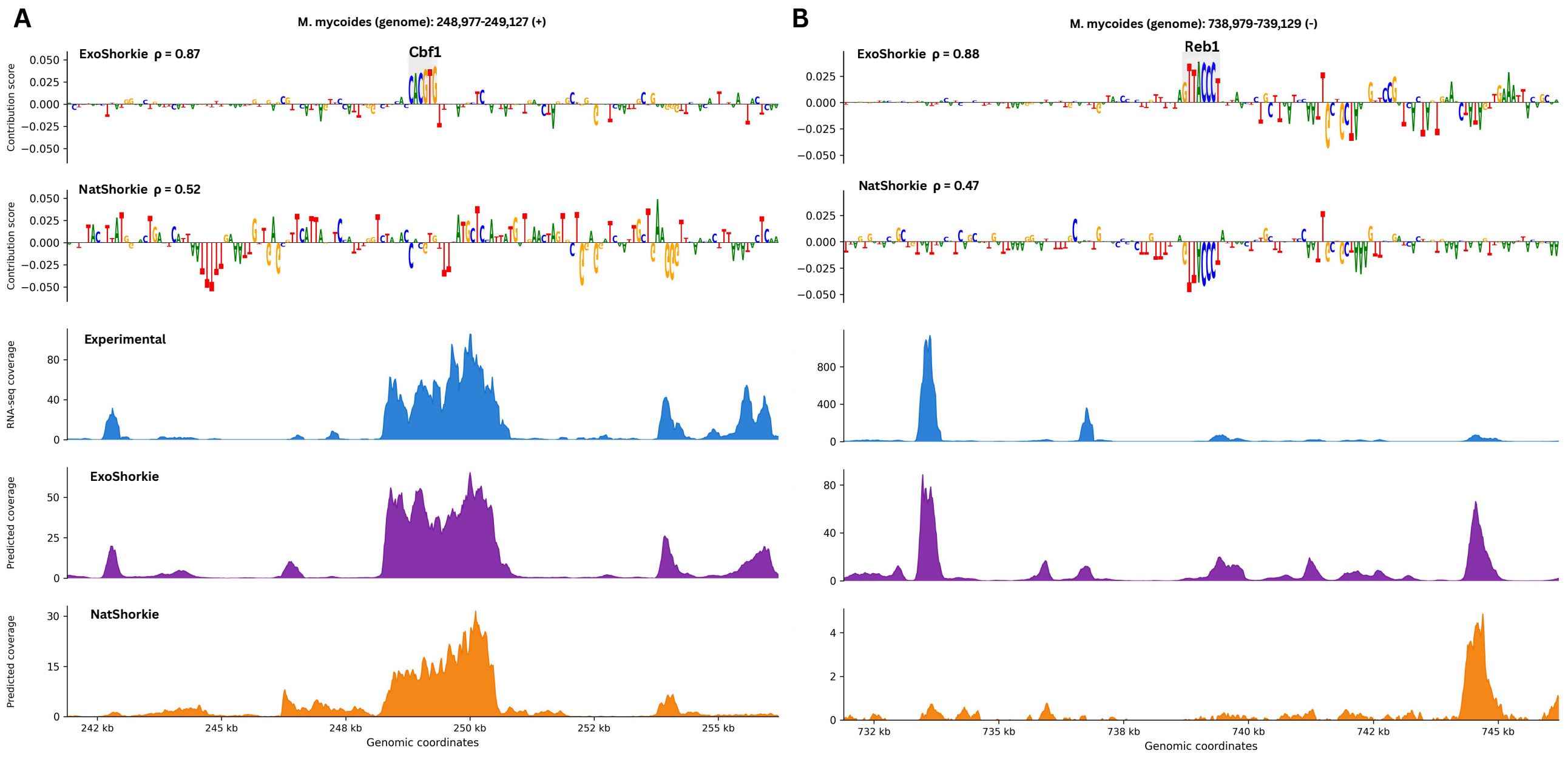

Fig. S11. ExoShorkie recovers canonical yeast TF motifs from ISM attribution maps on the exogenous *M. mycoides* genome that are diffuse or absent in NatShorkie. (A) Contribution scores from ExoShorkie (top) recover a Cbf1 motif that is not recovered by NatShorkie (bottom). (B) Contribution scores from ExoShorkie (top) recover a Reb1 motif that is not recovered by NatShorkie (bottom). For both loci, the Spearman correlation (ρ) between predicted and measured RNA-seq coverage in motif-centered windows is significantly higher for ExoShorkie. ExoShorkie's predicted coverage profile is more concordant with the true RNA-seq signal across the central 14,336 bp predicted region of the 16,384 bp window centered on each motif.

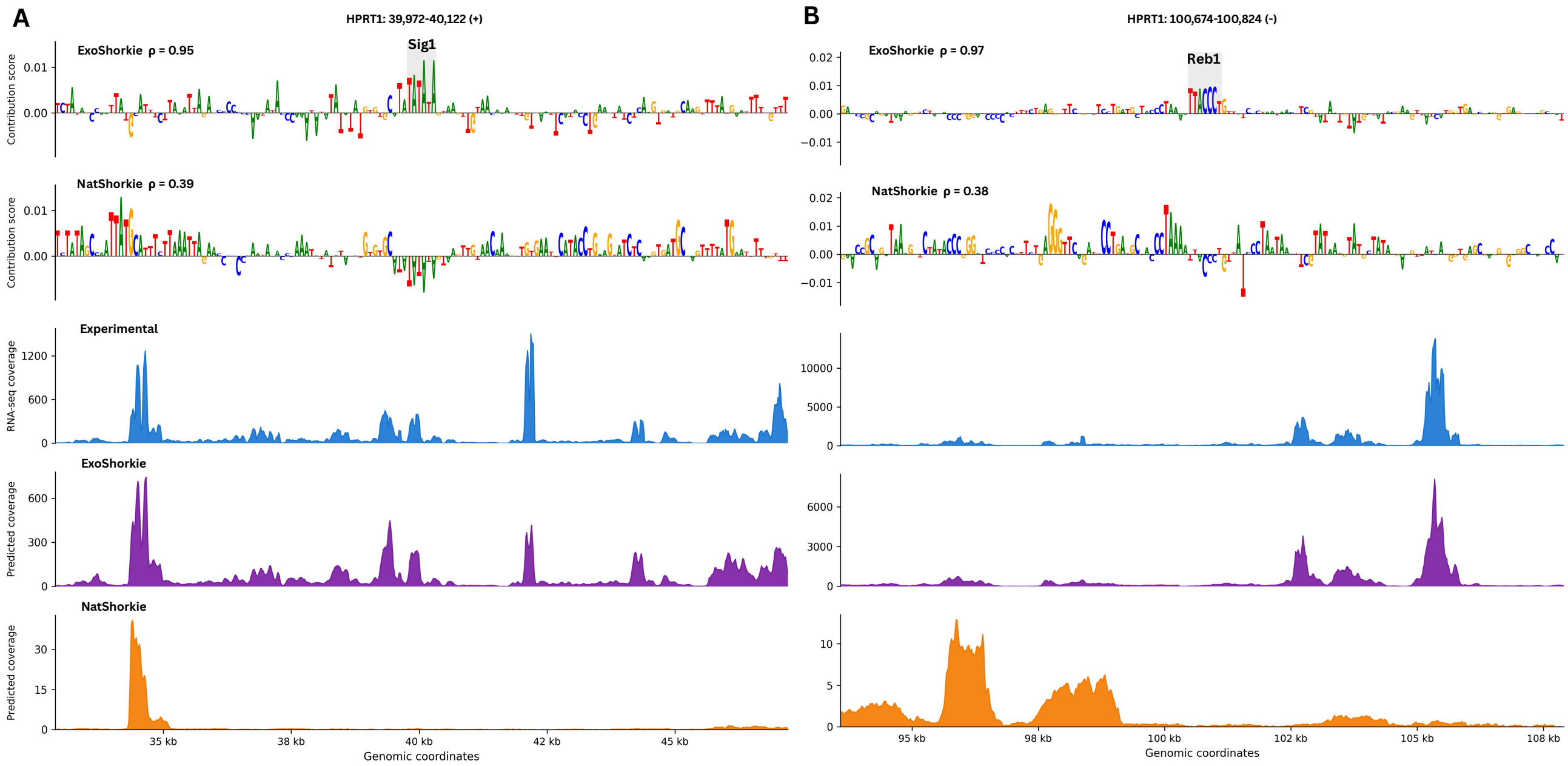

Fig. S12. ExoShorkie recovers yeast TF motifs from ISM attribution maps on the exogenous HPRT1 genome that are diffuse or absent in NatShorkie. (A) Contribution scores from ExoShorkie (top) recover a Sig1 motif that is not recovered by NatShorkie (bottom). (B) Contribution scores from ExoShorkie (top) recover a Reb1 motif that is not recovered by NatShorkie (bottom). For both loci, the Spearman correlation (ρ) between predicted and measured RNA-seq coverage in motif-centered windows is significantly higher for ExoShorkie. ExoShorkie's predicted coverage profile is more concordant with the true RNA-seq signal across the central 14,336 bp predicted region of the 16,384 bp window centered on each motif.

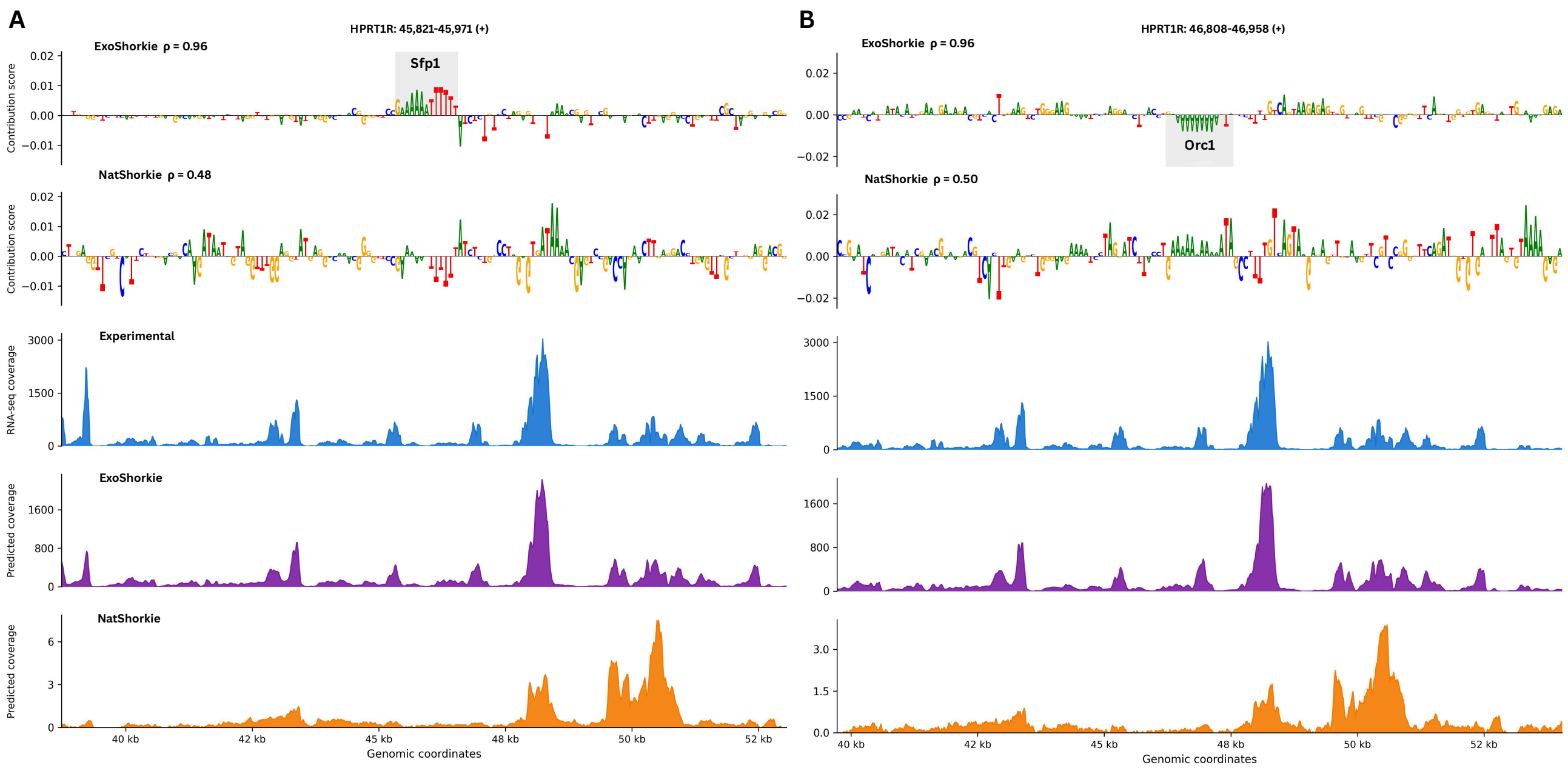

Fig. S13. ExoShorkie recovers yeast TF motifs from ISM attribution maps on the exogenous HPRT1R genome that are diffuse or absent in NatShorkie. (A) Contribution scores from ExoShorkie (top) recover a Sfp1 motif that is not recovered by NatShorkie (bottom). (B) Contribution scores from ExoShorkie (top) recover a Orc1 motif that is not recovered by NatShorkie (bottom). For both loci, the Spearman correlation (ρ) between predicted and measured RNA-seq coverage in motif-centered windows is significantly higher for ExoShorkie. ExoShorkie's predicted coverage profile is more concordant with the true RNA-seq signal across the central 14,336 bp predicted region of the 16,384 bp window centered on each motif.

References

Ž. Avsec, V. Agarwal, D. Visentin, et al. Effective gene expression prediction from sequence by integrating long-range interactions. *Nature Methods*, 18(10):1196–1203, 2021.

Ž. Avsec, N. Latysheva, J. Cheng, et al. Advancing regulatory variant effect prediction with AlphaGenome. *Nature*, 649(8099): 1206–1218, 2026.

K.-H. Chao, M. M. Magzoub, E. Stoops, et al. Predicting dynamic expression patterns in budding yeast with a fungal DNA language model. *bioRxiv*, 2025.

S. Gupta, J. A. Stamatoyannopoulos, T. L. Bailey, et al. Quantifying similarity between motifs. *Genome Biology*, 8(2):R24, 2007.

M. Pachkov, P. J. Balwierz, P. Arnold, et al. SwissRegulon, a database of genome-wide annotations of regulatory sites: recent updates. *Nucleic Acids Research*, 41(D1):D214–D220, 2012.

A. M. Ribeiro-dos Santos and M. T. Maurano. Iterative improvement of deep learning models using synthetic regulatory genomics. *bioRxiv*, 2025.

J. Schreiber. tangermeme: A toolkit for understanding cis-regulatory logic using deep learning models. *bioRxiv*, 2025a.

J. Schreiber. Tomtom-lite: accelerating Tomtom enables large-scale and real-time motif similarity scoring. *Bioinformatics*, 41(11): btaf577, 2025b.

O. Yaish and Y. Orenstein. F-MoDA: Efficient Fourier-based motif discovery in attribution maps. In *Machine Learning in Computational Biology*, pages 326–335. PMLR, 2025.
